# Results of the 3 Pillars Study (3PS), a relationship-based programme targeting parent-child interactions, healthy lifestyle behaviours, and the home environment in parents of preschool-aged children: A pilot randomised controlled trial

**DOI:** 10.1371/journal.pone.0238977

**Published:** 2020-09-17

**Authors:** Samantha Marsh, Rachael Taylor, Barbara Galland, Sarah Gerritsen, Varsha Parag, Ralph Maddison

**Affiliations:** 1 National Institute for Health Innovation, School of Population Health, University of Auckland, Auckland, New Zealand; 2 Department of Medicine, University of Otago, Dunedin, New Zealand; 3 Department of Women’s and Children’s Health, University of Otago, Dunedin, New Zealand; 4 Epidemiology and Biostatistics, School of Population Health, University of Auckland, Auckland, New Zealand; 5 Institute for Physical Activity and Nutrition, Deakin University, Burwood, Australia; Ghent University, BELGIUM

## Abstract

**Background:**

Early childhood is a critical period for the development of obesity, with new approaches to prevent obesity in this age group needed. We designed and piloted the 3 Pillars Study (3PS), a healthy lifestyle programme informed by attachment theory for parents of preschool-aged children.

**Methods:**

A 2-arm, randomised controlled pilot study was conducted to assess the effectiveness of 3PS, a 6-week programme involving a half-day workshop plus 6-week access to a study website. The programme was designed to promote routines around healthy lifestyle behaviours, including sleep, limited screen use, and family meals, within the context of positive, reciprocal parent-child interactions. Parents (n = 54) of children aged 2–4 years who regularly exceeded screen use recommendations (≥1 hour per day), were randomised to the 3PS programme (n = 27) or a wait-list control group (n = 27). Child screen time at 6 weeks was the primary endpoint. Frequency of family meals, parent feeding practices, diet quality, sleep, Child Routine Inventory (to assess predictability of commonly occurring routines), and household chaos were also assessed. Study data were collected online at baseline, 6 weeks, and 12 weeks via REDCap.

**Results:**

No group differences were observed for changes from baseline in screen time (primary endpoint), feeding behaviour scores, Child Routine Inventory scores, or total night time sleep duration at 6 and 12 weeks, although all measures improved in the hypothesised direction in the 3PS group. Compared with controls, the intervention group demonstrated significant improvements from baseline in household chaos scores (i.e. a reduction in chaos) and a number of measures of sleep outcomes, indicating improved sleep continuity. The programme was highly acceptable to parents.

**Conclusions and recommendations:**

A relational approach appears promising as a novel way to promote healthy lifestyle behaviours associated with the prevention of childhood obesity in children aged 2–4 years. A larger study is warranted.

## Introduction

Early childhood is a critical period for the development of obesity. Children who are overweight at 5 years of age are four times more likely to develop obesity between the ages of 5–14 years compared to children with a healthy weight [1]. Moreover, 72% of children who are obese when they are 5 years old demonstrate entrenched obesity, that is, obesity that is established and difficult to reverse [2]. Because obesity in early childhood is strongly associated with increased rates of premature death in later life [3], early obesity prevention strategies that target preschool-aged children are increasingly a focus [4, 5].

Obesity in preschool-aged children is linked with a number of modifiable lifestyle behaviours, including screen use, low levels of physical activity, poor diet, sugar-sweetened beverage consumption, and inadequate and/or poor quality sleep [6]. Programmes designed to prevent obesity aim to alter energy-related behaviours (ERBs) by modifying one or more of these behaviours [7]; however, these programmes are met with varying degrees of success [8, 9]. Common barriers to operationalising the recommendations provided in obesity prevention programmes have been identified. These include parents’ concerns over how to manage their child’s behaviour (e.g. tantrums over food) [10], lack of time and economic resources [11–13], cultural beliefs regarding perceptions of healthy body size [14, 15], parents’ unwillingness to acknowledge and discuss their child’s weight issues [16], and feeling overwhelmed by the chaos and hurriedness of daily life [17].

Providing parents with advice about healthy lifestyle guidelines, but not supporting them to overcome these barriers may increase parental stress and guilt [18], the likelihood of using strategies that pattern unhealthy behaviours in the long term [19], and parent-child conflict [20]. For example, asking parents to increase their child’s intake of vegetables may inadvertently put pressure on the parent to achieve this goal, resulting in use of counterproductive strategies, such as coercive feeding practices, which have been shown to decrease vegetable intake in the long term [21]. In the absence of understanding what is developmentally appropriate behaviour for a young child, parents may become overly fixated on the child’s behaviour (e.g. eating their vegetables), resulting in increased parental intrusiveness and greater child resistance [19]. Unfortunately, parents in food-insecure households may be more susceptible to adverse feeding behaviours, such as restrictive feeding and pressuring their child to eat [22], which are associated with overweight in children [23]. In order to avoid both unintended consequences of obesity prevention programmes and increasing inequities, new approaches are necessary.

It has been argued that a relational approach, that moves beyond behaviour change, may enable parents to respond more appropriately to their children’s ‘negative’ behaviours, as it encourages them to reconceptualise the child’s behaviour from that of non-compliance, to a developmentally appropriate demonstration of their sense of agency or need to feel connected to their parent [24]. This enables parents to switch focus from the child’s behaviour (i.e. the outcome), to processes by which healthy behaviours are fostered. Within a relationship-based approach, these processes may be defined as positive, warm, and mutually responsive parent-child interactions. When viewed through this lens, parents may be more likely to be responsive to the emotional needs of their child during health-related behaviours, rather than viewing the child’s behaviour as a demonstration of disobedience that requires parental intervention. As a result, they may be less likely to engage in adverse parenting behaviours, as they switch focus from the outcome (e.g. eating vegetables), to the process for developing healthy eating behaviours (e.g. positive parent-child interactions at the dinner table), with the understanding that the behaviour itself may not change immediately but that this is not an indication of failure or an adverse outcome. Ultimately, parents are encouraged to engage in positive parent-child interactions, which underlie the development of healthy attachments, with the goal of building trusting and safe relationships, which then become the context for promoting health-related behaviours.

This approach focusses on the potential bi-directional nature of causality between parent-child interactions and outcomes. In this model, the focus is on how parental practices affect the child’s behaviours and vice versa, resulting in a feedback loop whereby the actions and behaviours of both parent and child affect one another. Recently the bi-directional nature of parent-child interactions has been identified as an important, yet largely overlooked piece of the childhood obesity puzzle [25]. Current approaches to obesity prevention tend to focus primarily on the actions of the parents, that is, a unidirectional model; however, a bi-directional approach changes the focus to the dynamic nature of the parent-child dyad [26–28]. When there is dyadic mutuality between the child and parent, defined as the presence of warm and mutually responsive parent-child interactions, then interactions between both parties are more likely to be experienced as positive and rewarding and thus may encourage ongoing engagement in them [25, 29]. We hypothesise that this relational approach proposed for addressing fussy eating may also be extrapolated to other health-related behaviours, including sleep and screen use, which does not appear to have been examined previously.

An attachment-based, relational approach may also help address some of the common barriers encountered in childhood obesity prevention programmes. First, while placing the focus on the ‘outcome’ (e.g. adequate sleep, playing independently, or eating vegetables) may result in counterproductive parenting practices that interfere with development of healthy lifestyle behaviours in the long term, shifting the focus to the ‘process’ of developing healthy behaviours (e.g. positive parent-child interactions) may prevent engagement in adverse parenting practices. Second, by focusing on the parent-child relationship and attachment rather than body weight, it is not necessary for parents to identify their child as being at risk for overweight. Finally, it enables us to target aspects of the home environment that interfere with the parent-child relationship, including parental screen use [30] and a chaotic home environment [31–35], which in turn might reduce parental stress and feelings of hurriedness. In doing so, we may be able to promote a home environment that encourages parent-child connection, while reducing parental stress and the time and economic barriers identified as barriers to implementing healthy lifestyle recommendations [34, 36].

To this end, we designed and piloted the 3 Pillars Study (3PS), a healthy lifestyle programme for parents of preschool-aged children that promotes routines around healthy lifestyle behaviours, including sleep, limited screen use, and family meals, within the context of positive, reciprocal parent-child interactions. The programme identified positive parent-child relationships as the ideal context for promoting development of healthy eating, sleeping, and screen use habits. We hypothesised that this approach could potentially avoid the use of negative and counterproductive parenting practices, while addressing a number of the key barriers to implementing healthy lifestyle recommendations.

## Materials and methods

### Study design and participants

A 2-arm, randomised controlled trial (RCT) pilot study was conducted to assess the preliminary effectiveness of the 3PS study, in order to inform a larger trial. The protocol and content development manuscript has been published previously [37]. 3PS comprised of a half-day workshop and then subsequent access over a 6-week period to supplementary materials and information on a study website. Participants in the intervention group and the wait-list control group underwent study measures at baseline, 6 weeks, and 12 weeks. Following completion of the 12-week measurements, wait-list control participants were given the opportunity to participate in the 3PS programme. The Consolidated Standards of Reporting Trials (CONSORT) guidelines for reporting parallel group randomised trials were followed [38].

We recruited parent-child dyads between August 27, 2018, and September 6, 2018. Participants were parents or primary caregivers of children aged 2–4 years whose daily screen use exceeded current recommendations for this age group (i.e. ≥1 hour/day). Screen use was assessed during the screening process by asking the parent “Does your child regularly (on most days) use a screen for at least 1 hour per day?” Participants were aged at least 18 years, lived in the greater Auckland area, had access to the internet, were available to attend one half-day workshop, were able to provide electronic informed consent, and could speak and read English. Parents of children with known developmental problems or serious physical or mental illness were excluded due to the exploratory nature of the study.

Participants were recruited using social media (i.e. targeted Facebook advertising and posts). The advertisements linked to a study webpage where parents were provided with detailed information about the study and the contact details of a member of the research team. After contacting the research team, potential participants were provided with a verbal explanation of the study and emailed a copy of the Participant Information Sheet and Consent Form. If interested in participating, all potential participants were then asked to provide verbal consent to undergo screening over the phone. Eligible individuals were then sent an online link, where they consented electronically to participate in the study and completed a baseline questionnaire.

Participants were then randomly allocated to one of two groups (in a 1:1 ratio) using sequentially numbered sealed envelopes. The study statistician created a computer generated random sequence using block randomisation with randomly varying block sizes of two and four. The intervention commenced within 2 weeks of randomisation. 3PS was piloted in Auckland, New Zealand. Participants attended the workshop at the University of Auckland and all baseline, 6-week, and 12-week follow-up data was completed online. Study participants received $50 vouchers for completing the baseline and 6-week questionnaires, and a $20 voucher for completing the 12-week questionnaire. The study received ethics approval from the University Auckland Human Participants Ethics Committee (UAHPEC; reference 021311) and was registered with the Australian New Zealand Clinical Trial Registry (ACTRN12618000823279).

#### Intervention group

Parent-child dyads randomised to the intervention group participated in a half-day, face-to-face workshop at the University of Auckland, which was delivered by a trained facilitator. During the workshop, parents were given a Parent Handbook (S1 File), which provided an overview of the workshop and space to make notes. The 3PS programme aimed to promote mutually responsive, positive parent-child interactions at bedtime, mealtimes, and when restricting screen use/promoting free play. The programme was based on the Connecting Activities, Routines, and Environments (C.A.R.E) framework, which was developed for this study and has been reported in detail in our protocol paper [37].

Briefly, the framework provides a blueprint for creating routines that support the development of healthy lifestyle behaviours in the long term within the context of positive, reciprocal parent-child interactions [39–43]. This concept of creating a routine around fostering connection, and consequently the willingness of the child to cooperate, was vitally important as it helps to meet the four identified dyadic components for achieving mutually responsive orientation: (1) coordinated routines, (2) harmonious communication, (3) mutual cooperation, and (4) emotional ambience [26]. The core principle of the C.A.R.E framework is that a positive relationship between a child and their parent is the ideal context for building trust and a feeling of safety, and that a child needs to feel safe in order to sleep, eat, and play. Subsequently, messages around sleeping, eating, and reducing screen use were reframed so as not to focus on health outcomes, such as body weight. Instead, parents were encouraged to view these activities as opportunities to connect with their child and create daily routines and rhythms that help build a trusting relationship and meet their children’s need to feel safe and secure [44].

During the workshop, parents were first provided with a user-friendly, theoretical understanding of attachment theory and how attachment relates to child development and well-being [45–47]. They were then introduced to the C.A.R.E. framework, which included a discussion around the importance of positive parent-child interactions (i.e. ‘connecting activities’) for building a healthy parent-child relationship, and how routines and the home environment can either help or hinder these activities and, in turn, the relationship. Time was spent discussing what children find to be ‘disconnecting’, that is, things that can interfere with the quality of parent-child interactions, including always rushing around, being busy, and the parent being on their phone. Then parents explored activities that children find particularly ‘connecting’, including songs, storytelling, rough and tumble play, and affection. A comprehensive description of ‘disconnecting’ and ‘connecting’ activities can be found in S1 and S2 Files. Parents were encouraged to conceptualise their child’s challenging behaviours not as demonstrations of non-compliance or defiance, but rather a reflection of their need to feel more connected to themselves as the parent.

In the second part of the workshop each of the three ‘pillars’, including sleep, family meals, and free play, were introduced individually and explored using the theory from the first part of the day. For each health behaviour, parents were introduced to the importance of the behaviour with respect to child well-being and development, and what activities are particularly disconnecting and connecting for that specific behaviour. The C.A.R.E framework was then used to help parents design a routine around the health behaviour, which included removing disconnecting activities, and adding in more connecting activities [24, 48].

Given that parent-child interactions may be disrupted by aspects of the home environment, parents were also encouraged to identify and reduce barriers to connection within the home, including household chaos [31, 49–52] and their own screen use. Note, household chaos refers to the level of disorganisation, noise, and environmental confusion in the home, and has been associated with a range of adverse child outcomes. It has been suggested that household chaos impacts children by increasing levels of stress and distraction, interfering with children’s attention allocation and information processing skills, and compromising positive parent-child interactions and parental responsiveness [53].

Before thinking about how the environment might impact on the specific health behaviour, however, the facilitator read out two scenarios. Participants were invited to close their eyes and imagine the scenario in their heads. The first scenario involved a chaotic home environment. For example, a messy bedroom with toys all over the floor, bright lights overhead, and loud noises. The second scenario involved a less chaotic home environment. For example, a clean and tidy bedroom with soft lighting, the bed made with one or two favourite toys on it, and hushed tones. Participants were then asked to imagine which environment was more conducive to the specific health behaviour. Finally, at the end of each ‘pillar’ participants were asked to come up with a small change that they were going to make at home over based on what they had learned during the workshop.

After the workshop all participants were given access to a study website, which provided all the workshop content, for a period of 6 weeks. The study website content is provided in S2 File.

#### Wait-list control

Parents randomised to the wait-list control group were offered the 3PS programme, including both the workshop and 6-week access to the online content, after final follow-up was complete. Prior to this, they did not receive information about healthy lifestyle behaviours or promoting positive bi-directional interactions.

### Outcome measures

- Screen time was measured using four questions from the New Zealand Health Survey [54], where parents report the approximate amount of time their child spends watching TV or using ‘other’ screen devices during weekdays and on weekends. The response options were open-ended. Screen time was assessed as both continuous and binary (i.e. proportion of children meeting the screen time recommendations of less than 1 hour/day) variables. Screen time at 6 weeks follow-up was the primary endpoint.
- Family meal frequency was assessed using the question ‘How many days a week does your family usually sit together to eat any main meal?’, with possible responses ranging from 0 to 7 days. Four questions from the New Zealand Health Survey assessed servings of fruit, vegetable, fizzy drink, and fast food consumption (e.g. ‘On average, how many servings of fruit does [child’s name] eat per day?’. A serving size was explained and participants were given the following options: ‘They don’t eat vegetables’, ‘Less than 1 serving per day’, ‘1 serving per day’, ‘2 servings per day’, ‘3 servings per day’, ‘4 or more servings per day’, ‘Don’t know’, ‘Refuse to answer’.)
- To assess parental feeding practices, we also administered a modified version of the Feeding Practices and Structure Questionnaire [55], which assessed four domains of parental feeding behaviours, including using food as a reward for behaviour, rewarding for eating, persuasive feeding, and structured meal setting. For example, ‘I offer my child his/her favourite foods in exchange for good behaviour’, with the following response options: ‘Disagree’, ‘Slightly disagree’, ‘Neutral’, ‘Slightly agree’, ‘Agree’.
- The Brief Screening Questionnaire for Infant Sleep Problems (BISQ) Extended [56]–Adapted was used to assess sleep outcomes, which has been validated for toddler sleep in a New Zealand sample [57]. Sleep outcomes assessed included time the child goes to bed, how long it takes for the child to fall asleep, how difficult is if for the child to fall asleep, how many times the child wakes at night, how frequently the child wakes during the night, and how long the child sleeps for at night.
- Household chaos, which represents the level of disorganisation, environmental confusion, and background noise and distractions in the home [53], was assessed using the 15-item Chaos, Hubbub, and Order Scale (CHAOS) [58], and engagement in routines of daily life using the Daily Living Routines subscale of the Child Routine Inventory (CRI) [59].

Acceptability and feedback of the 3PS programme were assessed during the 6-week exit questionnaire using 5 open-ended questions with free text response options. Question included, ‘What did you like about the programme?’, ‘What could we change about the programme?’, ‘What did you learn from the programme?’, ‘What would you like to have learned that was not included in the programme?’, and ‘Would you recommend this intervention to another parent?’

Note, child bodyweight was not collected in this pilot study due to both resource constraints and the understanding that it is unlikely that such an approach would result in immediate changes in bodyweight.

#### Statistical analysis

As this was a pilot trial it was not powered to detect significant differences between groups; however, the sample provides sufficient data to ascertain recruitment and the direction and likely effect size for outcomes. Study data was collected in REDCap and all analyses were performed using SAS version 9.4. Baseline variables were summarised according to group and descriptive summary statistics provided. No significance testing was conducted on baseline measures as any differences between the groups at baseline could only have occurred by chance. Participants were not included in the analysis for an outcome if they were missing the outcome data on that measure. Change from baseline to 6 weeks and 12 weeks between the intervention and wait-list control groups were compared. The difference between groups in the binary outcomes were compared using Chi-squared tests. The distribution of continuous outcomes were assessed for normality. The change from baseline in the continuous outcomes was analysed using multiple linear regression modelling and adjusted for the baseline value. For non-normally distributed outcomes the median and inter-quartile range (IQR) data are presented, and the difference between groups were compared using the Wilcoxon two-sample test.

## Results

**Fig 1:**
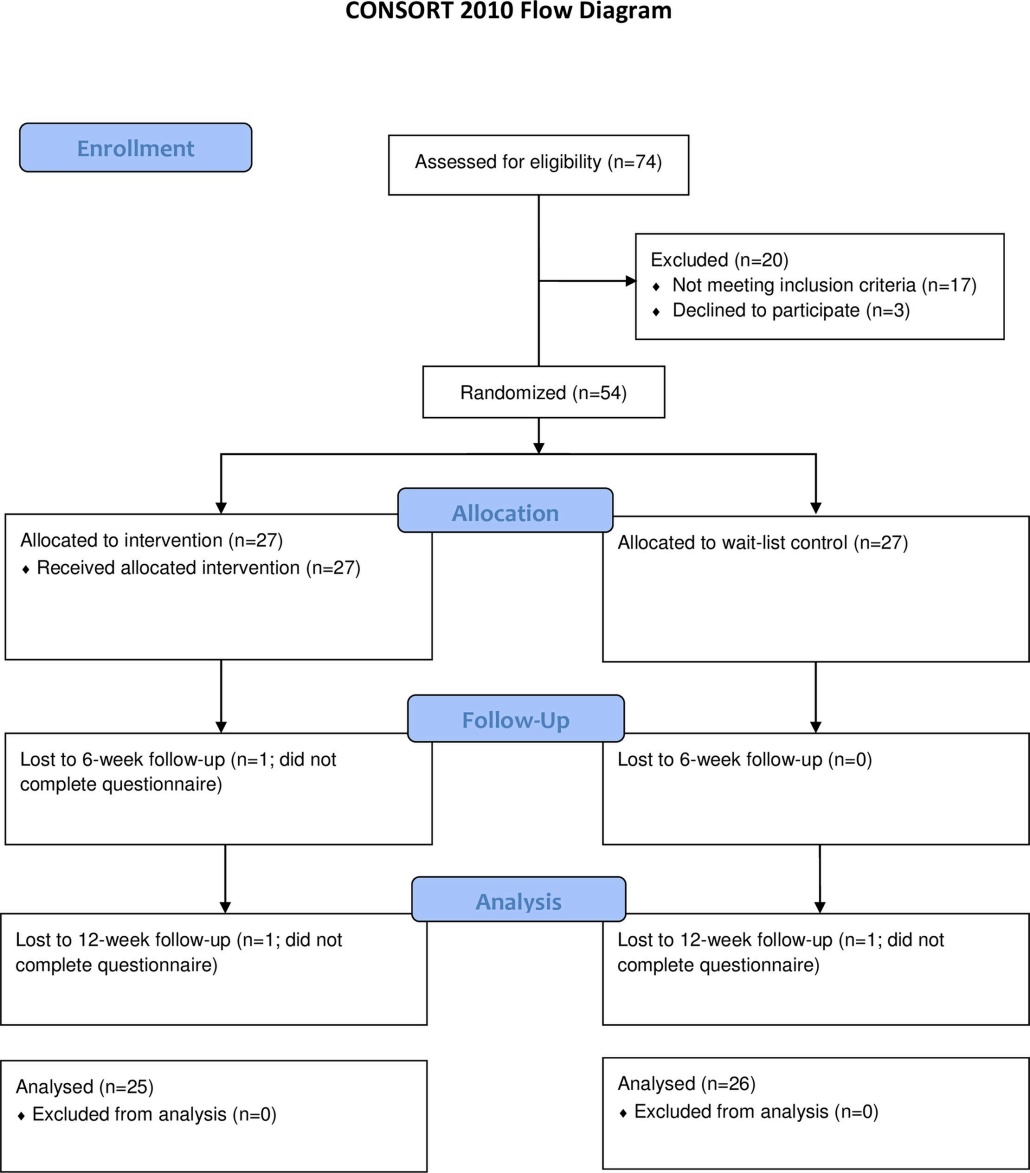
Flow of participants through the trial.

Fig 1 details the flow of participants in the study, with 54 participants randomised to the intervention (n = 27) or wait-list control (n = 27) groups. One participant was lost to follow-up at week 6 and a further two at week 12. Multiple attempts were made to contact these participants without success, as such, reasons for lost to follow-up were unknown. Overall, 94% (51/54) of participants completed final follow-up, and 96% of parents in the intervention group said they would recommend the programme to another parent.

Table 1 shows baseline characteristics for the study participants. The mean (SD) age of children in the study was 2.6 (0.7) years, and their caregiver was 35.9 (5.2) years of age. The majority of caregivers were the child’s mother (98.1%), NZ European (77.8%) and currently married (64.8%).

**Table 1:** Descriptive characteristics of the study population at baseline.

	Intervention	Control
	n	n
N	27	27
**Caregiver Characteristics**		
Gender (n, %):		
Male	1 (3.7)	0
Female	26 (96.3)	27 (100)
Prioritised Ethnicity (n, %):		
Māori	1 (3.7)	3 (11.1)
Pacific	0	1 (3.7)
Asian	2 (7.4)	3 (11.1)
Other	2 (7.4)	1 (3.7)
NZ European	22 (81.5)	19 (70.4)
Marital status (n, %):		
Married	19 (70.4)	16 (59.3)
Civil Union or living with partner	6 (22.2)	10 (37)
Divorced, separated or widowed	1 (3.7)	0
Never married (single)	1 (3.7)	1 (3.7)
Relationship to the child (n, %):		
Mother (biological or adoptive)	26 (96.3)	27 (100)
Father (biological or adoptive)	1 (3.7)	0
**Child Characteristics **		
Age (years), mean (SD)	2.6 (0.7)	2.5 (0.7)
Ethnicity (n, %):		
Māori	1 (3.7)	0
Pacific	0	2 (7.4)
Asian	1 (3.7)	1 (3.7)
Other	3 (11.1)	4 (14.8)
NZ European	22 (81.5)	20 (74.1)
Household child is living in (n, %):		
Original family (both biological or adoptive parents present)	25 (92.6)	25 (92.6)
Sole parent family	1 (3.7)	1 (3.7)
Living with extended family e.g. grandparents, relatives	1 (3.7)	1 (3.7)

No group differences were observed for changes from baseline in screen time, feeding behaviour scores, Child Routine Inventory scores, and total night time sleep duration at 6 and 12 weeks (Table 2); however, all measures improved in the hypothesised direction in the intervention group. Compared with the control group, the intervention group demonstrated significant improvements from baseline in CHAOS scores (12 weeks only; reflecting less household chaos), total time the child was awake during the night (6 weeks only), and longest stretch of time child was asleep during the night without waking (6 and 12 weeks) reflecting improved sleep continuity.

**Table 2:** Summary of continuous outcomes and P value for change from baseline between groups.

	Controls			3PS group				
Outcomes	Baseline	6 weeks	12 weeks	Baseline	6 weeks	12 weeks	P value* (6 weeks)	P value* (12 weeks)
**Sleep (mean, SD)**								
Median time during the night the child is awake (mins)#	10 (5, 30)	15 (5, 30)	5 (0, 15)	10 (0, 30)	5 (0, 10)	0 (0, 5)	**0.022#**	**0.171#**
Longest stretch of time the child is asleep during the night without waking up (hours)	7.4 (2.8)	8.1 (3.0)	7.7 (3.0)	8.8 (2.9)	10.2 (2.0)	10.2 (1.7)	**0.036**	**0.004**
Total time the child spends sleeping during the night (hours)	10.7 (1.0)	10.7 (1.0)	10.6 (1.2)	10.6 (1.1)	10.9 (0.7)	11.0 (0.6)	0.327	0.069
**Screen Use (mean, SD)**								
Screen time per day during the weekdays and weekends (hours)	1.8 (0.8)	1.8 (1.0)	1.9 (1.2)	1.6 (0.8)	1.4 (0.8)	1.5 (1.0)	0.243	0.374
**Parent feeding practices (mean, SD)**								
Uses food to reward behaviour score**	10.2 (3.0)	9.8 (3.2)	9.5 (3.2)	10.1 (3.1)	9.0 (3.6)	8.3 (2.9)	0.269	0.130
Uses food as a reward for eating score**	9.2 (3.9)	9.0 (4)	8.8 (3.6)	10.6 (3.4)	8.9 (3.8)	8.7 (3.2)	0.135	0.296
Persuasive feeding score**	18.4 (4.2)	18.5 (4.3)	17.4 (4.3)	18.5 (5.0)	17.3 (4.2)	16.6 (3.8)	0.067	0.141
Structured meal score	11.4 (3.0)	11.8 (2.8)	11.7 (2.7)	11.7 (2.5)	12.1 (2.2)	12.3 (2.5)	0.852	0.213
**Household (mean, SD)**								
Child Routine Inventory score	37.2 (4.8)	37.9 (4.3)	38.4 (3.7)	37.6 (5.5)	38.6 (5.5)	39.5 (4.6)	0.735	0.172
CHAOS score**	5.1 (3.5)	5 (3.2)	5.0 (3.2)	4.2 (2.4)	3.4 (2.4)	3.0 (2.1)	0.054	**0.002**

* P value for the between-group difference in the change from baseline (adjusted for baseline value)

# Data is non-normal so median and interquartile range are reported and Wilcoxon two-sample test used.

**A lower score is a more favourable outcome.

There was no significant difference between the groups at 6 and 12 weeks with respect to the proportions of children meeting screen time recommendations (<1 hour/day), meeting sleep guidelines (>11 hours/day), eating at least 2 servings of fruit per day, eating at least 2 servings of vegetables per day, and parents reporting bedtime as being ‘very easy’ or ‘easy’; however, again all measures improved from baseline in the hypothesised direction in the intervention group. Further, a significantly greater proportion of children in the intervention group versus control group were classified as only waking rarely during the night (i.e. 1–3 nights per month or less) at both 6 and 12 weeks follow-up (54% vs 22% and 35% vs 64% participants, respectively), and sleeping either ‘well’ or ‘very well’ at 12 weeks (84% vs 50%; Table 3).

**Table 3:** Summary of binary outcomes and P value for difference between groups (excluding missing).

	Controls			3PS group				
Outcomes	Baseline	6 weeks	12 weeks	Baseline	6 weeks	12 weeks	6 weeks P value*	12 weeks P value*
**Sleep**								
Bedtime is 'very easy' or 'somewhat easy'**	15 (55.5%)	12 (44.4%)	11 (42.3%)	15 (55.5%)	15 (57.7%)	17 (68%)	0.335	0.065
Proportion of children who don't wake at night during a typical week	8 (29.6%)	21 (77.8%)	16 (61.5%)	11 (40.7%)	11 (42.3%)	9 (36%)	**0.008**	0.068
Proportion of children who typically wake during the night ≤1–3 nights per month	9 (33.3%)	6 (22.2%)	9 (34.6%)	11 (40.7%)	14 (53.8%)	16 (64%)	**0.018**	**0.036**
Proportion of children who usually sleep 'well' or 'very well'***	12 (44.4%)	16 (59.3%)	13 (50.0%)	15 (55.5%)	19 (73.1%)	21 (84%)	0.288	**0.010**
Proportion of children who sleep at least 11 hours per night	16 (61.5%)	16 (59.3%)	15 (57.7%)	15 (55.6%)	17 (65.4%)	19 (76.0%)	0.646	0.166
**Screen Use**								
Proportion of children who meet screen use guideline of less than 1 hour	3 (11.1%)	5 (18.5%)	8 (33.3%)	2 (7.4%)	8 (30.8%)	7 (28.0%)	0.300	0.686
**Dietary behaviours:**								
2 or more fruit servings per day (%)	23 (85.1)	24 (88.9%)	22 (84.6%)	22 (81.4%)	24 (92.3%)	24 (96%)	0.670	0.172
2 or more vegetable servings per day (%)	17 (62.9%)	18 (66.7%)	19 (73.1%)	17 (62.9%)	20 (76.9%)	20 (80%)	0.407	0.560
Child eats meal with family at table each day 'often' or 'nearly always' (%)	18 (66.7%)	21 (77.8%)	18 (69.2%)	20 (74.1%)	21 (80.8%)	19 (76%)	0.788	0.588

* P value for the difference between groups

** Compared with ‘not easy or difficult’ or ‘somewhat difficult’ or ‘very difficult’

*** Compared with ‘fairly well’ or ‘fairly poorly’ or ‘poorly’ or ‘very poorly’.

## Discussion

The results of this pilot study provide preliminary evidence that a relationship-based intervention, which promotes positive parent-child interactions as the context for supporting development of healthy lifestyle behaviours, may improve a number of behaviours associated with childhood obesity in preschool-aged children. In particular, our results suggest that the 3PS intervention may be particularly beneficial for improving sleep outcomes in this population, with significant between-group improvements from baseline demonstrated for duration of nighttime awakenings, longest duration of uninterrupted sleep during the night, frequency of night-time awakenings, proportion of children who do not wake during the night, and proportion of children who sleep ‘well’ or ‘very well’. While significant between-group differences were not noted for other outcomes, except household chaos, all measures improved from baseline in the hypothesised direction in the 3PS group.

The 3PS programme is unique in that it focusses on promoting mutually responsive orientation between the parent and child, with this relationship being the context in which healthy lifestyle behaviours associated with obesity prevention are targeted. Consequently, rather than focussing on behaviour change methods to improve healthy lifestyle behaviours associated with overweight and obesity, namely diet, sleep, and screen time, we took an attachment-based perspective. This approach is in stark contrast to previous studies, which primarily focused on increasing parental knowledge and skills around child ERBs, and using behaviour change strategies [60]. Since attachment is a relationship-based construct, 3PS was designed to promote positive parent-child relationships, with a particular focus on times of the day when positive parent-child interactions may be challenged, including bedtime, screen use, and mealtimes. In order to achieve this, we did not focus on a specific behaviour (e.g. sleep) but instead on the importance of cultivating a positive parent-child connection and mutually responsive orientation at a specific time of the day (e.g. bedtime), while also identifying aspects of the home environment that may either facilitate or impede the parent-child connection at this time. To the best of our knowledge, 3PS is the first obesity prevention programme to specifically promote mutually responsive orientation, despite a persuasive argument that prevention attempts should be extended to target the bi-directional nature of the parent-child relationship [25].

While current literature focusses on the need to create positive emotional climates during meals and feeding [61], there is little on this topic with respect to sleep and screen use. This approach appears particularly valuable for addressing common sleep issues in this age group, which aligns with previous research in this area [62]. For screen use, we did not frame reducing child screen time as a goal of the programme, but rather reframed traditional messaging around reducing screen time. For emphasis, rather than focusing on reducing screen time to promote physical activity and prevent obesity, we discussed how screens interfere with the parent-child relationship and mutually responsive orientation. In particular, we focused on how screens interfere with child well-being through interruption of positive parent-child interactions, which aligns with recent findings from a cross-sectional study in 20 324 Chinese children aged 3–4 years old [63]. Finally, 3PS was also novel in that it focussed on reducing household chaos, which itself has been shown to be associated with child obesity [64], in addition to mediating the relationship between child sleep and bodyweight [65]. A chaotic home environment was discussed in terms of how it can interfere with a child’s ability to interact effectively with their parent, rather than how it is linked with obesity outcomes. In order to reduce household chaos, we discussed introducing family routines that support healthy lifestyle behaviours, for example family meals, reducing the level of chaos in the physical environment, and decreasing exposure to distractions created by screen devices.

A number of study strengths and limitations should be considered when interpreting the results from this study. Firstly, while we used a robust RCT study design, which we believed ethically managed the need for a control group, the sample size was small and thus the study was not powered to detect statistically significant differences in outcomes. As such, rather than being interpreted as a definitive demonstration of effectiveness, our findings are only an *indication* of effectiveness, but nevertheless encouraging for conducting a larger pragmatic RCT. Additionally, results from this pilot study can now be used to inform the design of a larger pragmatic RCT, enabling us to calculate the sample size and effect sizes. Further, the study population was largely homogenous and as such findings cannot be generalised to the population. Future research should be undertaken in priority groups, both for obesity in general and also in those with poor sleep, given preliminary evidence for the effectiveness of this approach with respect to sleep in particular. Importantly, we demonstrated improvements in sleep outcomes in a group that had not self-identified as having a child with a sleep issue. It will be important to establish whether findings from this study can be replicated in children with common, non-clinical sleep problems. Formative work must also be conducted to assess the feasibility and appropriateness of this approach in specific priority groups. A number of important outcomes were also not collected during follow-up, for example parental self-efficacy, the quality of parent-child interactions and parent-child relationship, and child bodyweight. Where possible, parent self-reported outcomes will also need to be objectively measured in a future trial. Finally, a future trial will also need to include an active control group, in order to strengthen the study design [66]. Given the nature of this approach, these outcomes would be vital in a larger RCT to assess programme impact on target behaviours and outcomes (while noting that improvement in long-term healthy lifestyle behaviours is unlikely to result in immediate change in body weight).

## Conclusions

The 3PS programme appears promising as a novel approach to promoting healthy lifestyle behaviours associated with the prevention of childhood obesity in children aged 2–4 years. We demonstrated that such an approach is highly acceptable to parents, and has potential to improve ERBs, in particular sleep. A larger study is warranted.

## Supporting information

S1 File (PDF)

S2 File (PDF)

S3 File (PDF)

S4 File (PDF)
