# Supplementary material for: Results of the 3 Pillars Study (3PS), a relationship-based programme targeting parent-child interactions, healthy lifestyle behaviours, and the home environment in parents of preschool-aged children: A pilot randomised controlled trial
Source: PLoS One. 2020 Sep 17;15(9):e0238977. doi: 10.1371/journal.pone.0238977 (PMC7498059; doi:10.1371/journal.pone.0238977)
Supplement: S1 File — (PDF) [file pone.0238977.s001.pdf]

The 3 Pillars Study

Parent Handbook

Investigators: Dr Samantha Marsh, Professor Ralph Maddison, Professor Rachael Taylor, Associate Professor Barbara Galland, Dr Sarah Gerritsen

C.A.R.E. Framework

- The aim of C.A.R.E. is to find solutions to living in a busy world in order to reduce the pressure and stress placed on children and their families.
- The framework focuses on encouraging connections between the parent and child while removing barriers to this connection.
- The framework encourages engagement in connecting routines, parenting behaviours, and environments.
- The workshop will focus on family meals, bedtimes, and play; however, it can be used at any time of the day (e.g. busy mornings).

THE 'ACTIVITY'

- Children thrive on routine
- When things are done the same way every day, children can relax knowing what is expected of them
- Routines help a child's internal rhythm align with the external rhythm of the day (e.g. they feel hungry when it is time for dinner and sleepy when it is time for bed).
- Routines and rituals are very connecting for young children.
- Routines can help the most challenging times of the day go smoother (including meals, bedtimes, and play/screen use).
- Healthy family routines can have dramatic effects on children's health, behaviour, well-being, and development.

THE 'RELATIONSHIP'

- Children often say 'no' when they feel disconnected from their parents
- This is a natural instinct that young children have – when they feel like they are being controlled or pressured by someone who they don't feel connected to, their instinct is to push back
- Parents shouldn't take it personally
- It is important to connect with our children at various times throughout the day. For example, first thing in the morning, before meals, after kindergarten etc.
- Page 4 of this handbook has a list of things that young children find disconnecting
- Page 5 of this handbook has a list of things that young children find connecting

THE 'ENVIRONMENT'

- Distractions and an overly stimulating environment interfere with child well-being
- This is because distractions and overstimulation may detract from interactions between the parent and child, and the parent-child relationship
- When there is too much stimulation or distractions, children end up filtering things out, including their parent's voice
- Screens, including those in the background, parent screen use, and child screen use, act as a barrier between the parent and child and distract from the relationship
- A simplified home environment with minimal clutter, not too many options (e.g. not too many toys/books), and low levels of background noise (e.g. no TV on in the background) can have a positive impact on your child
- Making small changes in the home environment is easy and can also improve parent's state of mind

Using the CARE Framework

- **Step 1:** Think about what is disconnecting for children
- **Step 2:** Design a basic routine and make sure connection/bridging is included in the routine
  - Keep it simple
  - Make sure it has a clear beginning and end
  - Think about what disconnects and try to avoid this
- **Step 3:** Make a plan about how to actively infuse connection into the routine
- **Step 4:** Design an environment that supports connection
- **Step 5:** Make a small achievable change

What children find *DIS*CONNECTING

1. Busyness and rushing
2. Feeling coerced or overly controlled by their parent
3. Too much stimulation or too many options
4. Too many questions
5. Inconsistency from parents
6. Bribes and rewards
7. Distractions (e.g. screens)

CONNECT & BRIDGE

Singing (songs, rhymes, verses, fingerplay, prayer, and blessings)

Movement (physical connection, touch, and rough and tumble play)

Imagination (magic and storytelling)

Love (appreciation)

Exaggeration (humour)

Using the 'Senses'

Using 'Sameness'

Bridging the separation

*S.M.I.L.E is adapted from 'Joyful Toddlers and Preschoolers: Create a Life that You and Your Child Both Love', by Faith Collins

*Concepts of connecting through Senses, Sameness, and using Bridging are adapted from 'Hold On to Your Kids: Why Parents Need to Matter More Than Peers', by Gordon Neufeld and Gabor Maté

TOY SIMPLIFICATION

Consider removing toys that are:

- Broken
- Developmentally inappropriate
- Fixed (e.g. narrow use)
- Too complicated, break easily, require batteries
- Highly stimulating (e.g. loud, screens, bright flashing lights)
- Inviting to violent play
- Multiples (too many of each)

Consider keeping toys that are:

- Loved
- Whole
- Inviting to the imagination
- Inviting to the sense of touch
- Visible
- Can be put away in 5 minutes

*Toy Simplification adapted from 'Simplicity Parenting: Using the Extraordinary Power of Less to Raise Calmer, Happier, and More Secure Kids', by Kim John Payne

EXTRA IDEAS FOR FAMILY MEALS

Revolving menus

This is when a series of menus is repeated over a specific period (e.g. 1 or 2 weeks). The menu is different each day of the cycle. At the end of the cycle, the cycle repeats from the start. For example, Sunday is roast night, Monday is pasta night. etc. Daily dinners are more streamlined and predictable, and often less stress for the cook. There is less discussion on what food will be eaten and in what quantity. This leaves more time for connecting. As the menu items are repeated, kids become more familiar with the meals. It also allows a rhythm to be introduced to meals at a different level (e.g. not just about having dinner at the same time each night, but also a routine around WHAT is for dinner each night). This can further be promoted by creating a routine about what foods are offered at the beginning and end of meals.

Add vegetables and fruit into the routine

Start every dinner with cut vegetables or a simple salad and finish with a fruit platter. When children become used to and familiar with this routine, they are more accepting of the fruit and vegetables. It also eases parents stress around what is eaten at meals.

Weekly meal planning

Saves money and reduces stress and busyness around meals. Consider sharing the meal preparation and cooking among adults in the household across the week – the same cook every night can be boring (especially for the cook!).

Plan for busy nights

Have frozen meals in the freezer and plan these for those nights when you know things will be a rush.

Repeat exposure

Kids may need up to 15 exposures before they trust a new food to even taste it. Then up to 32 tastes before they will like it. Don't give up. This is normal!

Think about texture and different ways of presenting foods

When introducing a new food, the child's brain acts like it has a series of 'doors' that need to be opened. The first door is texture – so you may need to 'hide' the texture of a new food. For example, start with the vegetable blended into a smoothie then slowly introduce its 'real' texture. Some children prefer vegetables raw or lightly steamed, mashed or roasted – try different preparation methods.

Pairing new foods with 'safe' foods

Always serve a 'safe' food (that your child knows and likes) when you introduce a new food, and make sure the 'new food' serving size is small while the 'safe' food serving is larger.

The power of physical hunger

A child who is physically hungry may be more receptive to new foods; avoid snacks and filling the child up on milk in the two hours before mealtimes.

Teach your child to eat mindfully

Babies are born with the ability to self-regulate their food intake. That means that they will eat when they are hungry and stop when they are full. Adults can override this ability by coaxing babies to “finish a bottle” or insisting that children “eat everything on the plate”. Remind your child to “listen to their body” and note the internal signals that they are hungry or full.

Vegetables can be given at breakfast

If you worry about vege consumption, try and get vegetables and fruits in earlier in the day. For example, offer a smoothie at breakfast or lunch – this may take the pressure off at dinner. And make vegetables and fruit visible at home and encourage them as snacks.

Take into consideration your child’s palate

The best meals are the ones that children eat. This doesn’t mean separate meals for adults and children, but is just a reminder that a child’s palate is highly sensitive and salt, acid, and bitter can be strong tastes for children.

When they just won’t eat, keep it positive

Remind them that they can’t eat until the next meal/snack. But keep this positive, e.g. “Does your tummy feel full? Remember that you can’t eat again until breakfast time. You might get hungry before then.” If they refuse to eat anything, or only a few mouthfuls, then the meal is over and the food should be taken away with no fuss.

Healthy eating behaviours last a lifetime

It can seem like hard work getting everyone to the dinner table, keeping the atmosphere peaceful, positive and fun, and making sure your child is offered a variety of healthy foods. Remember that it is worth it! Teens and adults are more likely to eat healthy food, eat breakfast and value family mealtimes if these behaviours were followed in their childhood.

Notes
