# Supplementary material for: Results of the 3 Pillars Study (3PS), a relationship-based programme targeting parent-child interactions, healthy lifestyle behaviours, and the home environment in parents of preschool-aged children: A pilot randomised controlled trial
Source: PLoS One. 2020 Sep 17;15(9):e0238977. doi: 10.1371/journal.pone.0238977 (PMC7498059; doi:10.1371/journal.pone.0238977)
Supplement: S2 File — (PDF) [file pone.0238977.s002.pdf]

The C.A.R.E Framework

CONNECTING ACTIVITIES

Children love having things done exactly the same way every day. These little rituals enable children to anticipate what will happen next, so they know how things go, and eventually they will be able to do them themselves. This helps children develop competence. Further, establishing these daily rhythms allow the child's internal rhythm to develop, so that when it is time to eat they feel hungry, and when it is time to sleep they feel sleepy.

On the other hand, when children don't know what is expected of them or when they are unsure of what is coming next, they feel disoriented and disconnected. A consistent rhythmical flow of basic routines throughout a child's day, particularly around meals rest, and play, helps strengthen the connection between the parent and child.

When daily activities are incorporated into routines and rituals, tension and confrontations at what can be the most challenging times of the day begin to melt away. As a result, instead of being disconnecting, these key moments can turn into moments of connection. This is particularly good news for busy parents – by implementing routines at a few key moments throughout the day, children can feel more connected with their parents.

CONNECTING RELATIONSHIPS

Often when we ask young children to do something our request is met with a forceful 'no'. This is confusing to parents, especially when we know our children can comply with such requests. So why is this? Why do they listen to us one day and meltdown at the same request the next? According to the book "Joyful Toddlers and Preschoolers", this is because when children say no, what they are actually saying is, "I don't feel as connected to you right now as I wish."

As it turns out, all children have a natural instinct to resist a request when they aren't feeling connected. This is important as young children easily fall out of connection with parents and caregivers throughout the day. This is perfectly normal and is not a reflection of parenting skills. The key to dealing with this is to understand what our children find disconnecting, and then find simple ways to re-connect when necessary.

What Children find Disconnecting

Busyness and rushing

- Young children do not respond well when we are constantly rushing around. When we are busy we are distracted, and when we are distracted we are less responsive to our children's requests and needs.

Feeling coerced or controlled by the parent

- Children have a natural instinct to resist us when they don't feel connected enough AND/OR when they feel like we are pushing our own 'will' and desires onto them.

- When we are too obvious about what *we* want, their instinct is to push back and try and control the situation, usually by saying ‘no’!

- For this reason, it helps to keep our emotions and desires under wraps a little bit. A perfect example is when children are being fussy at the dinner table. This can be very distressing for parents and parents often ‘show their hand’ – pushing and coaxing the child to eat. Children pick up on this and push back by refusing to eat. But if they don’t perceive that we care, then they can focus on their own will: “Am I hungry?” “Do I want to try this new food?”

Too much stimulation or too many options

- Young children are easily overwhelmed and when they feel overwhelmed they often can’t make a decision about what to do and then push back.
- Too much stimulation also detracts from parent-child interactions, so that children feel disconnected to us. As discussed above, when children feel disconnected from us, even when we make a simple request this can often be met with a ‘no’ or even a tantrum.

Too many questions

- Asking too many questions is very unsettling for kids as they feel safest when they perceive that we have all the answers. If we are constantly asking them what they want then they may take this to mean that we don’t know what we are doing. There are two important rules around giving options:

    - Avoid asking children questions about care-taking duties (e.g. food, bed etc.), such as ‘do you want to go to bed now?’, ‘what should I make you for dinner’? Children need to feel secure that we know how to take care of them.

    - It is good to give choices to children as this makes them feel like they have some control. However, it is best to only give two options, and both options should be agreeable to you. E.g. ‘would you like to wear your pink top or your green top today?’ Not, ‘what would you like to wear today?’

Inconsistency from parents

- This is similar to ‘asking too many questions’. Children feel safest when they believe we know what we are doing (even if we don’t!) and when they know what is expected of them.

- When parents are inconsistent with their own behaviour children perceive this as a weakness and an indication that we don’t know what we are doing. If a child knows that we will give in then they will quickly use this to their own advantage. For

example, if at mealtimes they know that if they don't eat their dinner we will offer them something else, then they will become very stubborn around food.

Bribes & Rewards

- Rewards are often recommended as a means to encourage toddlers and preschoolers to do something (e.g. using a sticker chart at bedtime). There are two problems with rewards and bribes:

    - They reveal our own desires and as we know, if a child is not actively connecting with us then they will push back – even if they want the same thing we want.

    - It reinforces that the reward is good and the thing we are trying to get them to do/eat/play with is bad. E.g. "eat your veges because they are good for you, otherwise there will be no dessert." In this instance, we are decreasing the value of veges and increasing the value of dessert.

- As a result, although rewards appear to work in the short term (when the child wants to get the thing you are rewarding them with), in the long term the child learns to push back against bribes and rewards and our requests.

Distractions

- One of the most disconnecting things we can do as parents is engage in activities where we are emotionally unavailable. For example, when we are on our phones, children often stop what they are doing and seek out our attention. This is because when we are on a screen we are not emotionally available to our children – if they ask us a question we either don't hear them or get annoyed.

- In essence, children perceive us to be 'connected' to our screen rather than with them

- Children respond better when we are emotionally available. This does not mean we need to stop everything we are doing though. For example, when we are doing household chores, we are not distracted – we can still talk to our children and interact with them. And we are much less likely to feel annoyed when our child interrupts 'chores' versus activities where we are physically available but not emotionally available (e.g. screen use, talking on the phone, reading a book).

- When children use screens, they connect with the screen which means they disconnect from us

What Children find Connecting

- Songs, rhymes, verses, fingerplay, prayer, and blessings
- Movement, physical connection, touch, and rough and tumble play
- Imagination, magic, and storytelling)
- Love and appreciation
- Exaggeration and humour)
- Using the senses: This involves connecting through sound (e.g. hearing parents voices in another room), sight (pictures on the wall of a parent), smell (e.g. parents perfume), and touch (e.g. rubbing a child's back).
- Using sameness (including role modelling): It is very comforting for young children to feel that they are the same as us. So talking about how mummy and daddy go to bed at night and do the same things as them helps a child feel safe. This is also where role modelling comes in (e.g. at mealtimes). Another way to connect through sameness is tell a story from your childhood that the child can connect to. E.g. when I was a little girl I used to love building blocks just like you.
- Bridging the separation
    - **Separation is alarming for a child.** This is why bedtimes and saying goodbye at kindy are often so difficult. Young children are not yet equipped to hold onto their 'connection' with you throughout the day. The best way to ease anxiety around periods of separation that are not avoidable is to bridge the separation.
    - **Bridging is all about putting the focus on the next connection**
    - Examples of bridging:
        - Giving your child something 'special' to hold onto that they can take to bed/with them that reminds them of you and how much you love them
        - Talk about what you are going to do together when you see them again e.g. a puzzle after kindy, a big cuddle in the morning
        - Fill them up with 'connection' before the separation! E.g. Try and build in connecting activities into your morning, before they go off to kindy, and at night, before they go to bed.
        - Put the focus on the next connection and make it tangible e.g. when the timer goes off I will come check on you, when I finish folding this basket of washing we can play etc.

CONNECTING ENVIRONMENTS

Too many distractions, including too much stuff, too many toys, and too much noise, interfere with almost every aspect of child well-being and development. Screen use in particular is highly distracting, whether it be a screen in the background, such as a TV being on in another room, parent screen use, or child screen use.

This is because distractions interfere with important interactions between the child and their parent. As a result, children find overstimulating and distracting environments disconnecting.

Experience shows that clearing away the clutter from children's home environments can build resiliency, balance family life, and create more space and time for the child and the parent. Within such an environment, children learn to be more creative, adaptive, and innovative – and it gives them the much needed space to play.

Parents can easily make small changes to the home environment that can positively impact on a child's behaviour – and their own mental health.

HOW TO USE THE FRAMEOWRK

Step 1: Think about what might be disconnecting you from your child

Avoid disconnecting activities and situations or make a plan about how these may be minimised.

Step 2: Design the basic routine and make sure connection/bridging is included in the routine

- Keep it simple: It is important to keep routines simple so that they don't take too much time out of the day – routines are meant to provide you with more time. Also, it is important that parents don't try to do too much at once.
- Make sure there is a clear beginning and end to the routine: This helps to ground children and let them know what is expected of them.

Step 3: Make a plan about how to actively infuse connection/bridging into the routine

Connecting and bridging help turn boring routines into special rituals that children find connecting.

Step 4: Create an environment that is connecting

- A peaceful bedroom
- A play sanctuary
- A nourishing and nurturing family table

Step 5: Plan a small change

NOTE: Be patient. These things may feel strange initially and unnatural – and your child may not respond well. However, as they get used to them, they will begin to follow along with less fuss.

SLEEP

BACKGROUND

Sleep is a challenge for almost all young families in New Zealand. Young children require much more sleep than adults to nourish them. Experts recommend around 12 hours of sleep per day for preschoolers. However, the average preschool-aged child today sleeps 9.5 hours so over the course of one week, this adds up to a sleep deprivation of 17.5 hours!

Tired toddlers and preschoolers have greater anxiety, demonstrate less joy, have poorer problem solving skills, and are more likely to get sick. In short, poor sleep impacts every aspect of child development and well-being. It is therefore hard to overstate the importance of sleep for young children.

In order to sleep, children must feel safe. This feeling of safety enables them to relax and rest. This is the focus of this section – helping a child feel safe at bedtime through a restful environment, connection with the parent/s, and a soothing bedtime routine they can rely on.

For children who have never been good sleepers or who are chronically sleep deprived, the process will take extra time and extra work. Be patient and focus on the ultimate goal.

USING THE CARE FRAMEWORK FOR SLEEP

Children need to feel safe, both physically and emotionally, in order to sleep. The most alarming thing for a young child is separation from their parent and the biggest separation a young child faces is saying good night at bedtime. Change the focus from the separation and put it on the connection.

STEP 1: WHAT DISCONNECTS AT BEDTIME

- Rushing around in the lead up to bedtime
- Organised activities that run late and lead to a later bedtime
- Too many options at bedtime e.g. how many books do you want?
- Bribes/rewards and punishments e.g. sticker charts, threats, taking things away from the child that they love (e.g. you won't be able to sleep with your favourite toy)
- Parental distractions e.g. parents on their phone during the bedtime routine

STEP 2: DESIGN THE BASIC ROUTINE AND MAKE SURE CONNECTION/BRIDGING IS INCLUDED IN THE ROUTINE

Keep it simple, set up anchor points. That is, make sure there is a clear beginning and end.

An example routine might look like the following:

- A simple dinner around 5:30 (experts recommend early dinners)
- Bath

- Brush teeth and make sure their nappy is fresh or that they have been to the toilet.
- Book or tell a story. Only one or two. Do the same every night so the child gets used to not asking for more books. If they know they are only allowed one then after a little time, this will become their routine that they rely on and they will stop asking for more books.
- Connect/Bridge
- Lights out/Sleep
- Morning – reconnect after the separation

STEP 3: ACTIVELY INFUSE CONNECTION INTO THE ROUTINE

Suggestions for connecting and bridging at bedtime

- *Use a timer. Say that you will put the timer on and come and visit them every xx number of minutes. Initially, this may need to be only every 30 seconds at a time. But once your child figures out that you are coming back to check on them (and you need to make sure that you do), they will begin to relax. As time goes by just saying you will be back soon is enough for the child to relax and drift off to sleep. = Bridging*
- *Let them know what you are doing while they are in bed e.g. I'm going to wash the dishes now/feed the cat and then will come check on you = Bridging*
- *Give them something physical of yours to hold onto that will connect you through the night, such as a piece of clothing = Senses*
- *Tell them that you sleep in a bed just like theirs and that you will be next door keeping them safe until the morning = Love + Sameness*
- *Spray your perfume/aftershave into the room = Senses*
- *Tell them that you will leave something special in their room during the night that they can find in the morning (e.g. a sticker on their bed, a drawing you have made) = Bridging*
- *Put a picture of yourself on the wall by their bed, and tell them it is you looking over them = Love + Bridging*
- *Tie an invisible string between their bed and your bed. This attaches your beds together at night. Tell them that whenever they wake up during the night they can pull on the string and you will know it is them = Bridging + Imagination*
- *Sing them a lullaby = Singing*
- *Rub their back or hold their hands still with your hands (if they have 'busy hands' that keep them awake) = Movement/Touch + Love*
- *Make up a story but only tell part of it…tell them you will finish the story tomorrow = Bridging*
- *Tell them that you will meet them in dreamland tonight, and in the morning tell them a lovely story about what you did in your dream with them the night before = Imagination + Bridging*

- *Leave the door open at bedtime and tell them to listen out for the sounds of your footsteps or your voice. This is to remind them that you are close. After they fall asleep you can close the bedroom door if you like = Senses*
- *Talk about something special you want to do together the following day. It doesn't have to be big e.g. a cuddle first thing in the morning = Bridging + Love*
- *Tell them before bed that you will check on them throughout the night. And then tell them in the morning how you went into their room through the night and gave them a kiss and told them you loved them = Bridging + Love*

STEP 4: DESIGN AN ENVIRONMENT THAT SUPPORTS CONNECTION

Here are some ideas:

1. Remove screens from the bedroom and from the pre-bed routine (including parent and child screen use)
2. Remove noise from background screens
3. Make sure their bed is made, and doesn't have too many things on it
4. Tidy toys into baskets so the child can't see them at night. One or two simple toys on a shelf is fine
5. Think about soft lighting
6. Not too many brightly coloured pictures on the wall. Opt for warm tones
7. The bed should always have one or two favourite soft toys
8. Make sure their pyjamas are comfortable
9. Make sure they aren't too warm or too cold
10. Blackout curtains and if they have a nightlight make sure it is very soft and warm

PLAY

BACKGROUND

Too often play is pushed aside to make room for screen time or structured activities. This may happen because we have become preoccupied with things like physical activity or education, or when we fail to understand that these other activities don't provide the same benefits of play.

No activity promotes a child's psychological well-being quite like play. This is because during play children find their voice and begin to understand who they are as individuals. Children can be creative in play, and safely express their emotions and deal with whatever is upsetting them.

So what is true play? According to Dr Gordon Neufeld:

1. True play is not work, and therefore there should never be a focus on outcomes.

2. True play is never for real, and therefore should not have consequences.

3. True play is expressive, meaning that it should come from within the child and the child should not be passive in the activity. This is why screen use is almost never considered true play.

There are a number of different types of play, including social play, outdoor play, and independent play. While this course focuses on independent play, we recommend that you make time each day for outdoor play, as children who spend time outdoors ever day behave better when indoors, sleep better, and are less picky at mealtimes.

Independent play is often more difficult to encourage, especially in children who frequently use screens or are used to having their parents play with them. By creating a routine of daily independent play, parents are laying the foundation for building creativity, problem-solving skills, and resiliency.

USING THE CARE FRAMEWORK FOR PLAY

STEP 1: WHAT DISCONNECTS AT PLAYTIME

- Rushing around during the day so there isn't enough time for play (both outdoor play and independent play)
- Organised activities that we think are substitutes for play and so we use to replace play
- Too many options at playtime e.g. too many toys
- Rewards and a focus on outcomes e.g. drawing a picture for a person rather than just drawing for fun. Being overly concerned with 'learning opportunities'.
- Focusing on doing the activity the 'right' way rather than just enjoying it e.g. kicking a ball around the backyard versus learning to play soccer
- Parental distractions e.g. parents on their phone while the child is playing – this is the fastest way to distract a child away from independent play

Design the basic routine and make sure connection/bridging is included in the routine

1. Set aside time each day when you will encourage play
2. Set up a place to play *before* they play e.g. set some toys up in a 'scene' that encourages play
3. Connect
4. Move child towards toy area and play briefly with them
5. Begin to blend into the background
6. Stay connected/bridge the divide
7. When the child comes back to you, re-connect. The younger a child is the more frequently they will need to re-connect with you. Expect this.
8. Redirect them again to independent play (as above).

Step 3: Make a plan about how to actively infuse connection/bridging into the routine

- Before play, use 'Connect'.
- When 'blending into the background' – Bridge the separation. Try singing or humming so they can still hear you when you are out of the room. Try sitting in the room with them doing something boring so they can see you. For many parents, if you've spent the last couple of years 'playing along' with your child, this transition step may be difficult.
- Avoid activities where you are emotionally unavailable but physical available (e.g. screen use)
- Under 3's: Children under the age of three are often preoccupied with connecting so play is typically done in short bursts with adults and others nearby. Expect this. The child may need to come back and actively connect with you before being moved gently back to independent play.
- Older kids: When they become more independent and want to venture out on their own, they are more likely to get 'lost in play' for increasing periods. By the time a child is 5 years of age, they should ideally be able to play for extended periods – on their own and with others.

Step 4: Design an environment that supports connection

Why we should consider simplifying the play environment - *adapted from Simplicity Parenting*

1. Too much stuff leads to too many choices.
2. The number of toys your child sees and has access to can be dramatically reduced
3. Research shows that as you decrease the quantity of toys and clutter, you increase your child's capacity for paying attention and deep play.

4. Too many toys invites emotional disconnect and a sense of overwhelm – especially when the toys are loud or screen-based.

5. The child given less and less complexity will cultivate true powers of attention. The opposite is true when toys are overly prescriptive (e.g. a toy toaster that you can only really do one thing with)

6. Children's play flourishes when we let it, rather than make it, happen – this is why we need to leave children alone to play independently.

7. Too much stimulation causes sensory overload. Adjust the volume of sounds and light in your house by simplifying toys and minimizing interference from screens.

Design a play sanctuary

1. Undertake a toy simplification process

2. Toys displayed so children can easily see them

3. Get rid of the toy box - two things tend to happen with a toy box:

    - Everything gets dumped out onto the floor and is too overwhelming for the child to play with

    - It is ignored. This is because children as old as 5 years don't form a plan to play with a toy, they only play with something when they see it and they aren't overwhelmed by too many options and distractions.

4. Toys should be 90% child 10% kid (i.e. the child needs to use their imagination to play with the toy)

Family Meals

BACKGROUND

A preference towards healthy foods and healthy eating behaviours in young children is cultivated at the family table. Family meals provide parents with the opportunity to model healthy eating behaviours and connect with their children. However, a number of things get in the way of positive mealtime experiences. This is because, while parents are responsible for what, when, and how much food is offered to their child at mealtimes, it is the responsibility of the child to decide if and how much they eat. Unfortunately, there is often disconnect between what parents want and what children do. This can be very stressful for parents and leads to disconnecting behaviours.

Yet we know when children feel disconnected or like they are being coerced, they resist our requests, tantrum, and say “no”!

For this reason, it is so important to focus on positive interactions and connections in the lead up to, and during, meals. Also, easing some parent anxieties may be helpful. Remember:

- Missing meals will not harm your child. If they choose not to eat what you have provided, don’t make a fuss about it.
- Young children can self-regulate their energy intake from food. Trust them.
- Pickiness is normal and will pass
- Family mealtimes (eating together) are important, but will look different for each family.

For busy families, it may also help to ‘reimagine’ the family meal.

- The family meal can be at breakfast or lunch – it doesn’t have to be dinner
- The family meal doesn’t mean both parents have to be there – just one adult is fine
- If it is too early to eat dinner for you, just serve yourself a tiny portion of the meal your child is eating

New foods can be fun and exciting but also distressing for children. Therefore they need to feel safe and calm at mealtimes. Further, it is important to ‘connect’ new food with foods that are already familiar and liked by the child – try serving an unusual food alongside foods they know and like, or on a platter so they can help themselves (you can model trying the new food and give them the power the choose).

USING THE CARE FRAMEWORK FOR FAMILY MEALS

Children need to feel safe, both physically and emotionally, in order to eat. Change the focus from the food and eating and put it on the connection. Connecting is a great way to keep the child from moving into her “fight, flight, freeze and refuse” brain.

STEP 1: WHAT DISCONNECTS AT FAMILY MEALS

- Rushing around before the meal to get dinner prepared.
- Not sitting at a table. Eating around a table focuses the activity on conversation and connection, not just the food.
- Rushing the meal. Sit at the table for at least 20 minutes at dinnertime (but not longer than 40 minutes if children are no longer eating).
- Making it about your own will, including getting emotional around food, pressuring kids to eat, overtly controlling your child and food.
- Overwhelming a child, particularly around new foods.
- Too many options at meals/questions.
- Bribes/rewards and punishments. Also, using foods to soothe your child when they hurt themselves or are upset.
- Parental distractions. Make sure there are no screens at the table or in the lead up to the meal.

STEP 2: DESIGN THE BASIC ROUTINE

Remember: A routine around food sets a child’s internal body clock – so when it is dinnertime they feel hungry.

An example routine might look like the following:

1. Set the table (ask children to help if they are old enough)
2. Connect before the child sits down
3. Dinner starts around 5:30 (experts recommend early dinners)
4. Serve dinner once everyone is sitting properly in their chairs
5. Connect during the meal
6. Everyone to stay at the table until the meal is over (but they do not have to eat). Young children will get up and start to move away – gently bring them back to the table and state that at mealtimes we sit and talk while eating.
7. Children to say thank you for dinner or ask to be excused from the table once dinner is over
8. Dishes cleared from table (older kids can help with this)

STEP 3: MAKE A PLAN ABOUT HOW TO ACTIVELY INFUSE CONNECTION/BRIDGING INTO THE ROUTINE

- Cut vegetables into fun shapes, design food on a plate to be fun e.g. make a smiley face = Exaggeration (humour)
- Eat the food yourself (but DON'T talk about it), be obvious that you are enjoying it = Sameness (role modelling)
- Light a candle at the beginning of the meal = Senses
- Say a thank you verse or thank the chef = Love
- Hum a familiar song while dishing out the food or feeding the child = Singing
- Tell a funny story from your day = Exaggeration (humour)
- Pretend that the fork is heavy and difficult to get into your mouth = Exaggeration
- Tell a story and then stop when you get to an exciting part and say "take a bite" – and everyone has to take a bite = Imagination
- Swoop the child up like an airplane before putting them in their chair = Movement

STEP 4: DESIGN AN ENVIRONMENT THAT SUPPORTS CONNECTION

Design a nourishing family table

1. Soft lighting
2. Make sure the table and the room is clear of clutter
3. Light a candle
4. Remove all media from the room e.g. TVs, computers, phones etc.

EXTRA TIPS

Revolving menus: This is when a series of menus is repeated over a specific period (e.g. 1 or 2 weeks). The menu is different each day of the cycle. At the end of the cycle, the cycle repeats from the start. For example, Sunday is roast night, Monday is pasta night. etc. Daily dinners are more streamlined and predictable, and often less stress for the cook. There is less discussion on what food will be eaten and in what quantity. This leaves more time for connecting. As the menu items are repeated, kids become more familiar with the meals. It also allows a rhythm to be introduced to meals at a different level (e.g. not just about having dinner at the same time each night, but also a routine around WHAT is for dinner each night). This can further be promoted by creating a routine about what foods are offered at the beginning and end of meals.

Add vegetables and fruit into the routine. Start every dinner with cut vegetables or a simple salad and finish with a fruit platter. When children become used to and familiar with this routine, they are more accepting of the fruit and vegetables. It also eases parents stress around what is eaten at meals.

Weekly meal planning: Saves money and reduces stress and busyness around meals. Consider sharing the meal preparation and cooking among adults in the household across the week – the same cook every night can be boring (especially for the cook!).

Plan for busy nights: Have frozen meals in the freezer and plan these for those nights when you know things will be a rush.

Repeat exposure: Kids may need up to 15 exposures before they trust a new food to even taste it. Then up to 32 tastes before they will like it. Don't give up. This is normal!

Think about texture and different ways of presenting foods: When introducing a new food, the child's brain acts like it has a series of 'doors' that need to be opened. The first door is texture – so you may need to 'hide' the texture of a new food. For example, start with the vegetable blended into a smoothie then slowly introduce its 'real' texture. Some children prefer vegetables raw or lightly steamed, mashed or roasted – try different preparation methods.

Pairing new foods with 'safe' foods: Always serve a 'safe' food (that your child knows and likes) when you introduce a new food, and make sure the 'new food' serving size is small while the 'safe' food serving is larger.

The power of physical hunger: A child who is physically hungry may be more receptive to new foods; avoid snacks and filling the child up on milk in the two hours before mealtimes.

Teach your child to eat mindfully: Babies are born with the ability to self-regulate their food intake. That means that they will eat when they are hungry and stop when they are full. Adults can override this ability by coaxing babies to "finish a bottle" or insisting that children "eat everything on the plate". Remind your child to "listen to their body" and note the internal signals that they are hungry or full.

Vegetables can be given at breakfast: If you worry about vege consumption, try and get vegetables and fruits in earlier in the day. For example, offer a smoothie at breakfast or lunch – this may take the pressure off at dinner. And make vegetables and fruit visible at home and encourage them as snacks.

Take into consideration your child's palate: The best meals are the ones that children eat. This doesn't mean separate meals for adults and children, but is just a reminder that a child's palate is highly sensitive and salt, acid, and bitter can be strong tastes for children.

When they just won't eat, keep it positive: Remind them that they can't eat until the next meal/snack. But keep this positive, e.g. "Does your tummy feel full? Remember that you can't eat again until breakfast time. You might get hungry before then." If they refuse to eat anything, or only a few mouthfuls, then the meal is over and the food should be taken away with no fuss.

Healthy eating behaviours last a lifetime: It can seem like hard work getting everyone to the dinner table, keeping the atmosphere peaceful, positive and fun, and making sure your child is offered a variety of healthy foods. Remember that it is worth it! Teens and adults are more likely to eat healthy food, eat breakfast and value family mealtimes if these behaviours were followed in their childhood.
