# Supplementary material for: Results of the 3 Pillars Study (3PS), a relationship-based programme targeting parent-child interactions, healthy lifestyle behaviours, and the home environment in parents of preschool-aged children: A pilot randomised controlled trial
Source: PLoS One. 2020 Sep 17;15(9):e0238977. doi: 10.1371/journal.pone.0238977 (PMC7498059; doi:10.1371/journal.pone.0238977)
Supplement: S4 File — (PDF) [file pone.0238977.s004.pdf]

Study Protocol

The 3 Pillars Study: Routines and rituals to promote child well-being in busy families – A Pilot Study

Investigators: Dr Samantha Marsh, Professor Ralph Maddison

Table of Contents

Research Summary ---- *3*

Study Characteristics and Objectives ---- 4

Background ---- 4

Aims and Objectives ---- 6

Research Design and Methods ---- *6*

Study design ---- 6

Recruitment and sample size ---- 6

Eligibility ---- 7

Measures ---- 7

Intervention ---- 8

Randomisation ---- 10

Data Analysis ---- 10

Ethical considerations ---- *11*

Administrative section ---- *11*

Data confidentiality and security ---- 11

Publication policy ---- 11

Reimbursement ---- 11

Dissemination of Results ---- 11

Funding source ---- 12

Significance: ---- *12*

References ---- *13*

RESEARCH SUMMARY

**Title**	The 3 Pillars Study: Routines and rituals to promote child well-being in busy families – A Pilot Study
**Short Title**	3 Pillars Study
**Acronym**	3PS
**Aims**	To pilot the Three Pillars Study (3PS), a programme for busy parents of toddlers to encourage engagement in health-promoting family routines known to reduce the risk of childhood overweight and obesity.
**Objectives**	1. To investigate the preliminary effectiveness of the intervention on toddler sleep, screen use & play, diet, engagement in family routines, and the home environment.<br>2. To assess the preliminary acceptability of the intervention content to parents of toddlers and pre-schoolers<br>3. To inform the design of a feasibility study to investigate the feasibility of conducting a similar study on a larger scale
**Methods**	A two-arm, randomised control trial (RCT) design. Sleep, diet, screen use, and engagement in family routines outcomes will be collected, in addition to participant feedback on the programme.
**Study Centre**	National Institute for Health Innovation, Tamaki Campus, University of Auckland.

Study Characteristics and Objectives

Background

Childhood overweight and obesity remains one of the most pervasive and challenging public health issues facing New Zealand, and as such is a priority objective for the Ministry of Health. Almost 30% of all toddlers in New Zealand are overweight or obese, with higher rates in Maori (42.9%), Pacific (56.5%), and the most deprived (42.3%)[14]. This is concerning, given that weight status in the very early years is one of the strongest predictors for adult overweight[13]. In New Zealand, there has been growing interest in the way policy impacts on obesity, particularly within the food environment[16-21]; however, the social environment also influences childhood obesity[4-6].

Over the past 150 years life has been speeding up[1-3]. The modern environment is dominated by screen-based media (e.g. smartphones, iPads, and laptop computers), which allow people instant accessibility to entertainment, food, and each another[24], resulting in a quicker pace of life. This change in the speed of life has also been accompanied by an increase in household chaos, which is characterised by high levels of background stimulation in the home, lack of family routines, absence of predictability and structure in daily activities, and an overly fast pace of family life[1,2].

While a quicker pace of life is often associated with innovation, technology, and societal progression, there are also likely to be significant adverse effects, particularly within the family unit where household chaos has increased[24]. This may be particularly important when considering overweight and obesity in young children. With respect to physical activity, young children require a slower pace of life to ensure adequate development of physical literacy[10]. This is important, given that gross motor skills largely develop in infancy, with proficiencies tracking into later childhood and adolescence[27,28]. Deficiency in these skills is related with adverse effects on adiposity and physical activity levels[29]. When young children are not given adequate opportunities to play, this can negatively impact on their development of gross motor skills, which in turn may translate into a greater preference for sedentariness in childhood and adolescence. Unfortunately, free play in childhood has decreased in recent years, with parents reporting a ‘harried pace of life’ as a primary driver of this change[11], in addition to increased access to, and use of, mobile media devices.

Similarly for diet, early childhood represents a critical period of life during which taste preferences and eating behaviours are programmed and remain relatively stable throughout the lifetime[30,31]. Yet it is during these early years that children are first introduced to ‘fast’ foods i.e. pre-prepared, easily accessible infant and toddler foods, which are characterised by comparatively higher levels of salt and sugar compared with homemade wholefoods. One of the primary reasons parents cite for feeding young children these foods is lack of time[31]. Further, toddlers require time at meals to develop healthy eating behaviours (including avoiding picking eaters and developing preferences for fruit and vegetables[31]). Meals should therefore not be rushed, but should provide enough time for children to explore new foods, and familiarise themselves with new textures and flavours. Unfortunately, the amount of time people spend eating, where eating is their primary focus, has been decreasing, with the proportion of time spent eating while engaged in another activity, such as watching television, increasing between 1975 and 2006 (from 19%-22% to 43%-48%)[12]. This is concerning for children, given that eating in front of screens has consistently been shown to significantly increase energy intake from food[32].

Toddlers require adequate time to develop gross motor skills[10], healthy sleep patterns[22], and positive eating behaviours[31]. Deficiencies in each of these behaviours have been linked with childhood obesity[33]. Unfortunately, time allocated to these behaviours has been decreasing in recent years[11,12]. An intervention built on a foundation of slowing family life down, by reducing household chaos and promoting healthy family routines, may therefore help support development of healthy lifestyle behaviours in young children, which in turn may help prevent overweight and obesity in later childhood.

Such an intervention would target three key areas to promote patterning of healthy lifestyle behaviours in toddlers.

1. Free play and screen use: Toddlers have an innate desire to be physically active[34]; however, barriers in the modern environment restrict natural movement, including not providing enough time during the day for free play, restraining children in highchairs, car seats, and prams, and placing young children in front of screens for entertainment. Given toddlers' natural desire to be active, promoting free play and movement in toddlers may be achieved through removal of barriers to activity, rather than requiring parents to *encourage* physical activity.
2. Diet: Early childhood represents a critical period during which feeding behaviours and taste preferences are set. An intervention to slow family life down would encourage participation in regular family meals, removal of distractions from the feeding environment (e.g. screens), allocation of time for children to experiment with new foods, and promotion of quality parent-child interaction. In particular, a number of parenting practices, such as restrictive feeding and coercive feeding, have been linked with long-term adverse dietary behaviours in children, whereas positive parenting practices, such as role modelling, have been linked with development of positive dietary behaviours in children. Further, screens have repeatedly been shown to increase energy intake in children by distracting them from physiological signals of satiety[32], as such, ensuring screens are removed from meals would encourage self-regulation of energy intake, rather than overeating due to distraction.
3. Sleep: Adequate sleep hygiene is important in maintaining a healthy bodyweight. In very young children, a major threat to sleep is overstimulation, primarily from screens[35], lack of bedtime routines, and inconsistent parenting at bedtime. As such, the use of screens, particularly in the pre-sleep period would be a target for intervention, in addition to helping parents create routines that promote sleep.

This approach to intervention has potential to be sustainable and equitable

1. It does not involve the purchase of expensive toys or electronic devices. Indeed, the Slow Parenting movement (Simplicity Parenting), which is concerned with reducing household chaos and promoting healthy routines, asserts that the home environment that children are exposed to be pared back.
2. It does not require busy parents to allocate more time to their children, as it focusses on quality versus quantity. For example, it is about the quality of positive parent-child interactions rather than the quantity of time a parent spends each day with their child. Quality parent-child interactions can be cultivated within the context of family routines, such as positive family meals and child bedtime routines.
3. Parents with adequate resources and access to knowledge are already starting to simplify the environment their young children are exposed to (including the removal of screens)[41]. Yet young children of parents with lower levels of education, and those in minority groups are at greater risk for screen exposure, suggesting a social

gradient in screen-use behaviours even at a very young age[47]. In New Zealand, higher sedentary screen usage has been reported in Maori[48] and Pacific[48] children compared with other ethnicities, and it is therefore proposed that the burden of screen use in the very young may fall more heavily on these groups. As such, the increased accessibility, affordability, and availability of screens may actually be increasing disparities in health outcomes associated with screen use[47]. The proposed approach therefore has potential to promote more equitable health outcomes for young children by empowering parents to understand the importance of reducing household chaos, providing more opportunities for free play, and spending more time developing healthy eating and sleep behaviours.

Aims and Objectives

The aim of this research is to explore how reducing household chaos and encouraging engagement in positive family routines may promote development of healthy lifestyle behaviours that reduce the risk of overweight and improve well-being in young New Zealand children.

Findings will be used to inform the design of larger feasibility and effectiveness trials.

There are five main objectives of the study:
1. To assess the study design
2. To assess the study measures
3. To ensure the recruitment criteria and recruitment strategy are sufficient for larger trials
4. To assess preliminary effectiveness of the intervention
5. To gather feedback from the participants and trainer(s) about the programme

Research Design and Methods

Study design

A two-arm, 6-week RCT pilot study, with final follow-up at 12 weeks, will be conducted to assess the Three Pillars Study (3PS). Intervention participants will attend one half-day workshop and then have access to a supplementary website with more information for a period of 6 weeks. Both the intervention group and wait-list control group will undergo study measures at baseline, 6 weeks (at the end of the programme) and 12 weeks (final follow-up). After final follow-up data collection has been completed at 12 weeks, the control group will be offered the intervention, that is, participation in the half-day workshop and access to the study website for 6 weeks.

Recruitment and sample size

We aim to recruit 50 participants, 25 in the intervention group and 25 in the wait-list control group. Social media (i.e. Facebook) will be used for recruitment purposes. After participants contact the research assistant, an explanation of the study will be given to them and they will be sent the PIS/CF. If the potential participant expresses they are interested in participating, then the research assistant will ask for verbal consent to screen them. If they agree, then they will be screened by the research assistant on the phone. If they are eligible

to participate, they will be sent a link to the baseline questionnaire. Before completing the baseline questionnaire, they will be asked to provide electronic consent to participate in the study. Once they agree to participate, they will be able to complete the baseline questionnaire. After the baseline questionnaire is complete, the research assistant will randomise the participant to either the intervention group or the wait-list control and inform them of their randomisation group (more details on the randomisation process are provided below).

Completion of the baseline questionnaires and randomisation will take place during the 2 weeks prior to the first workshop. Participants in the intervention group will be asked to complete their 6-week and 12-week questionnaires 6 weeks and 12 weeks after the date of the workshop they attend, respectively. Three workshop dates will be offered on three consecutive Saturdays. Participants in the wait-list control group will complete their 6- and 12-week questionnaires 6 and 12 weeks from the date of the first workshop, respectively.

ELIGIBILITY

Inclusion criteria:

- Parent or primary caregiver aged at least 18 years of age with a child aged between 2 years and 4 years who exceeds guidelines for TV/screen use (i.e. >1 hour/day)
- Currently lives in Auckland and can attend one half-day workshop
- Has access to the internet
- Able to provide electronic informed consent, and can speak and read English

Exclusion criteria:

- Parent of a child with a serious physical or medial illness or known developmental problem (e.g. sleep apnoea, feeding aversions, autism)

MEASURES

All measures will be taken at baseline, 6 weeks (i.e. end of the programme) and 12 weeks (i.e. final follow-up) in both the intervention and control groups, unless stated otherwise.

Baseline (baseline only)

- Child: age, sex, ethnicity
- Caregiver: ethnicity, relationship to child, education, household size

Screen time/Activity

Screen time (primary endpoint): Assessed using four questions from the New Zealand Health Survey[3]. Parent-report of time (in hours) child spends watching TV or using 'other' screen devices during weekdays and on weekends. An average score for TV and 'other' screen time will then be calculated and the proportion of children meeting international guidelines (<1 hour per day for children aged 2-4 years) will be calculated. Screen time at 6 weeks is the primary endpoint for the study.

Diet

Nutrition questions: Four questions from the New Zealand Health Survey investigating number of fruit and vegetable servings, fizzy drink consumption, and fast food consumption, and one question from the Family Routines Inventory, investigating number of days per week that the family sit together to eat the main meal. One question from GUiNZ investigating frequency of family meals.

Feeding Practices and Structure Questionnaire[4] (modified): Seventeen questions investigating four domains of parental feeding behaviours: Reward for Behaviour, Reward for eating, Persuasive feeding, and Structured meal setting.

Sleep

Brief Screening Questionnaire for Infant Sleep Problems (BISQ) Extended[5] – Adapted: A series of parent-reported outcomes investigating nocturnal sleep duration, night wakings, method of falling asleep, sleeping arrangements, bedtime rituals, and parental Interventions. Stand-alone questions (i.e. no overall score is given).

Parent and Home Environment

Child Routine Inventory (CRI[6]; Daily living routines subscale only): 11 parent-reported questions investigating engagement in standard routines in the daily life of toddler. Scores are summed with higher scores indicating greater frequency of routines.

Chaos, Hubbub, and Order Scale (CHAOS)[7]: A single score is derived from 15 questions investigating parent's report of home characteristics. This single score is created by obtaining a simple sum of responses for the 15 items. The true or false responses are scored so that a higher score represents more chaotic, disorganised, and hurried characteristics of the home.

Exit Questionnaire (6-week follow-up only; intervention group only)
Acceptability and feedback: 5 open-ended questions about what the participants liked and disliked about the programme, and what they would keep the same and/or change.

Exit Interviews (6-week follow-up only; intervention only)
Around 6-10 participants from the intervention group will be asked to participate in an exit interview. The exit interview will be conducted by a trained research assistant on the telephone or face-to-face at a community venue at 6 weeks and will take approximately 20 minutes. Participants will be asked to read an additional PIS and provide informed consent before participating in the interview.

Note, we are attempting to improve health behaviours, the home environment, and parenting behaviours that are associated with overweight and obesity in later childhood and adulthood. Therefore, while the ultimate goal is to prevent overweight and obesity, we do not expect to see changes in bodyweight over the study period, as such, we will not be measuring anthropometrics.

INTERVENTION

The intervention content has been informed by trainings in the slow parenting/Simplicity Parenting[8-10] approach, household chaos research, parent focus groups, expert opinion, and an end-user panel. The 3 Pillars Study (3PS) intervention will consist of a half-day, group-

based workshop with parents of toddlers and preschoolers. A community worker, who will undergone extensive training, will deliver the content of the workshop in a community venue. The following outline will guide the workshop.

1. Introductions and welcome
2. A brief group exercise where parents/caregivers will discuss what they expect to get out of the workshop
3. Meaningful routines and rituals – what the research shows; why these are important to child health and well-being; how everyday stressors can impact on behaviour and health; how routines and rituals can help families deal with every stressors
4. The three pillars to health in busy families – simple solutions to overcoming barriers to engaging in healthy behaviours in toddlers
    a. Diet – this section will be focused on family meals. It will include a discussion around the importance of family meals, creating a meaningful routine around meals, positive parenting behaviours at mealtimes that have been shown to encourage development of long-term healthy eating behaviours in toddlers (e.g. role modelling, repeated offering of novel foods), and how to overcome fussiness at mealtimes.
    b. Play – this section will focus on what true play is, why it is important, and how parents can build time for free play into the daily routine. Given that screen use is not considered true play, and that it is a barrier to natural physical activity in toddlers, there will also be a discussion around screen use.
    c. Sleep – this section will focus on routines around nighttime sleep and day naps, and solutions to common sleeping issues at this age using a developmental, attachment framework.

    Each of the three pillars will be introduced in the same format:

    1. Why Simplify?
    2. Simplifying the Environment
    3. Simplifying the Relationship
    4. Simplifying the Activity

    These sections will follow the same format as the website, with more details outlined below.

    Note: the workshop will cover common challenges that many parents experience on a daily basis. However, if any parents identify more deeply ingrained or clinical issues, then they will be referred to their GP. In particular, the BISQ sleep questionnaire can be used to screen for clinical sleep problems that may require further investigation by a health professional.

After the workshop, participants will be given a study pack. The study pack will include a weekly meal planner magnet for the fridge, a candle for family dinners, a New Zealand cookbook that focuses on fussy eating in this age group, and a picture book to encourage reading to the child.

The workshop will be supplemented by access to the study website. The website will require a login and will be made available to intervention participants as soon as they have completed the workshop. The website will have the following pages:

1. 'Home' – this will be the landing page and will serve as an introduction to the study
2. 'Routines and Rhythms' – this page will provide an overview of the approach, i.e. slowing down the pace of home and family life, and will include general information around how reducing household chaos can positively impact on many aspects of children's health and well-being.
3. 'The 3 Pillars' – this will be divided into three sub-pages, which can be navigated to via a dropbox from the menu. The three pages, which will be entitled 'Eat', 'Play', and 'Rest' will all follow a similar format.
    i. Why Simplify? A brief overview of the topic (diet, play/screen use, and sleep), including some scientific information and why the activity is important to child well-being
    ii. Simplifying the Environment Practical solutions for simplifying the environment as it relates specifically to the topic. For play, this might be minimising toys, for meals, this might be ensuring the dining table is clear of clutter, and for sleep, this might be ensuring the bedroom and bed are.
    iii. Simplifying the Relationship This section will provide advice around constructive parent-child interactions around the activity. For example, for meals, this might mean not talking about food or nutrition at mealtimes and dealing with fussy eating. For sleep, this might include advice about how to interact with a child at bedtime and dealing with a child who will not stay in bed.
    iv. Simplifying the Activity Simple solutions and resources will be provided around how to create a routine around the activity. This might include meal planning, implementing a 7-day revolving menu, bedtime routines etc.
    v. Links and access to resources will also be provided.
4. 'About the Study' – this page will include the Participant Information Form and the Consent Form
5. 'Contact Us' – this page will include a contact phone number and email address for participants to contact the principle investigator

Randomisation

Participants that have consented and completed the baseline questionnaire will be randomly allocated to groups using sequentially numbered opaque sealed envelopes. Participants will be allocated to one of two groups in 1:1 ratio using block randomisation with varying block sizes of two and four.

Data Analysis

Baseline characteristics will be summarised using descriptive statistics. Continuous variables will be presented as mean and standard deviation (SD). Categorical variables will be presented as frequency and percentage. All statistical tests will be two-sided and maintained

at a 5% significance level. Categorical variables will be reported as n and %. Simple chi-squared analyses will be used to evaluate the treatment effect on the proportions adherent to screen use guidelines after 6 weeks, with estimation of relative risks, 95% confidence intervals and two-sided p-values.

A trained research assistant will conduct interviews. Interviews will be recorded (with permission) and transcribed verbatim. A general inductive thematic approach will be followed, which allows research findings to emerge from multiple readings of the raw data. NVivo9 software will be used to manage the transcripts and facilitate the analysis process, and to identify themes and categories.

ETHICAL CONSIDERATIONS

Ethical approval has been received from the University of Auckland Human Ethics Committee (021311).

ADMINISTRATIVE SECTION

DATA CONFIDENTIALITY AND SECURITY

Information will be kept confidential in keeping with the obligations set out in the Privacy Act 1993, the Health Information Code 1994 and Section 22B to 221 of the Health Act 1956. Data will be entered, stored and backed-up in a secure manner on a server at the National Institute for Health Innovation.

Data will be stored securely at password protected NIHI computers for 6 years. Only the research team will have access to these data.

PUBLICATION POLICY

There are no restrictions on publications.

REIMBURSEMENT

Participants will receive $50 vouchers after completing the baseline and 6-week follow-up assessments, and a $20 voucher after completing the 12-week follow-up questionnaire. This is to reflect the amount of time participants will have to dedicate to completing the assessments. Given this is a pilot study we are interested in assessing a range of potential outcomes using a variety of measures, which we anticipate taking between 20 and 30 minutes each at baseline, 6 weeks, and 12 weeks. Those participating in the exit interview will receive an additional $20 Westfield voucher.

DISSEMINATION OF RESULTS

The results will be presented in a written report and shared with stakeholders. Every attempt will be made to publish the findings in a scientific journal and/or present them at an academic conference.

FUNDING SOURCE

This study is funded by a Health Research Council Explorer grant.

SIGNIFICANCE:

Weight status in the very early years is one of the strongest predictors for adult overweight[13]. Given that 28.7% of all New Zealand toddlers are overweight or obese[14], unique ways of targeting them for the primary prevention of obesity are needed. This research aims to incorporate aspects of the 'Slow' framework and Slow/Simplicity Parenting into an intervention to improve behaviours associated with overweight in later childhood, including diet, physical activity, and sleep, and the home environment and parenting. This research fits within the Health Research Council's Research Investment Stream "Health and Wellbeing in New Zealand", and aligns with the Government's focus on childhood obesity, and the National Science Challenge of 'A Better Start', as it aims to improve the potential of very young New Zealanders by ensuring their health and developmental needs are not compromised by a fast-paced lifestyle during their formative years. If successful, these approaches will be more widely promoted for use by communities throughout New Zealand.

References

1. Wachs TD, Evans GW. Chaos in context. 2010.
2. Ackerman BP, Brown ED. Physical and psychosocial turmoil in the home and cognitive development. 2010.
3. Ministry of Health. Content Guide 2016/17: New Zealand Health Survey. In: Health Mo, ed. Wellington2017.
4. Jansen E, Mallan KM, Nicholson JM, Daniels LA. The feeding practices and structure questionnaire: construction and initial validation in a sample of Australian first-time mothers and their 2-year olds. *International Journal of Behavioral Nutrition and Physical Activity.* 2014;11(1):72.
5. Sadeh A. A brief screening questionnaire for infant sleep problems: validation and findings for an Internet sample. *Pediatrics.* 2004;113(6):e570-e577.
6. Sytsma SE, Kelley ML, Wymer JH. Development and initial validation of the Child Routines Inventory. *Journal of Psychopathology and Behavioral Assessment.* 2001;23(4):241-251.
7. Matheny AP, Wachs TD, Ludwig JL, Phillips K. Bringing order out of chaos: Psychometric characteristics of the confusion, hubbub, and order scale. *Journal of Applied Developmental Psychology.* 1995;16(3):429-444.
8. Payne KJ. *The Soul of Discipline: The Simplicity Parenting Approach to Warm, Firm, and Calm Guidance-From Toddlers to Teens.* Ballantine Books; 2015.
9. Payne KJ, Ross LM. *Simplicity parenting: Using the extraordinary power of less to raise calmer, happier, and more secure kids.* Ballantine Books; 2010.
10. Honoré C. *Under pressure: Rescuing our children from the culture of hyper-parenting.* Hachette UK; 2010.
